# Patent Review (2017–2020) of the Keap1/Nrf2 Pathway Using PatSeer Pro: Focus on Autoimmune Diseases

**DOI:** 10.3390/antiox9111138

**Published:** 2020-11-17

**Authors:** Dionysios V. Chartoumpekis, Chun-Yan Fu, Panos G. Ziros, Gerasimos P. Sykiotis

**Affiliations:** 1 Service of Endocrinology and Diabetology, Lausanne University Hospital, and Faculty of Biology and Medicine, University of Lausanne, 1011 Lausanne, Switzerland; dchart@upatras.gr (D.V.C.); panos.ziros@chuv.ch (P.G.Z.); 2 Division of Endocrinology, Department of Internal Medicine, University of Patras, 26504 Patras, Greece; 3 Department of Pathology and Pathophysiology, Zhejiang University School of Medicine, Hangzhou 310058, China; fcy1019@zju.edu.cn

**Keywords:** nuclear factor erythroid 2-like factor 2 (Nfe2l2), oxidative stress, text mining, patent landscape, autoimmunity

## Abstract

Research on the antioxidant pathway comprising the transcription factor nuclear factor erythroid 2-related factor 2 (Nrf2) and its cytoplasmic inhibitor Kelch-like ECH-associated protein 1 (Keap1) is ever increasing. As modulators of this pathway have started to be used in clinical trials and clinical practice, Nrf2 has become the subject of several patents. To assess the patent landscape of the last three years on Nrf2 and evaluate the main fields they refer to, we used the web-based tool PatSeer Pro to identify patents mentioning the Nrf2 pathway between January 2017 and May 2020. This search resulted in 509 unique patents that focus on topics such as autoimmune, neurodegenerative, liver, kidney, and lung diseases and refer to modulators (mainly activators) of the Nrf2 pathway as potential treatments. Autoimmunity emerged as the main theme among the topics of Nrf2 patents, including a broad range of diseases, such as systemic sclerosis, systemic lupus erythematosus, multiple sclerosis, inflammatory bowel diseases, Hashimoto’s thyroiditis, etc.; however, there was a dearth of experimental support for the respective patents’ claims. Given that chronic inflammation is the main element of the pathophysiology of most autoimmune diseases, the majority of patents referring to activation of Nrf2 as a method to treat autoimmune diseases base their claims on the well-established anti-inflammatory role of Nrf2. In conclusion, there is strong interest in securing intellectual property rights relating to the potential use of Nrf2 pathway activators in a variety of diseases, and this trend parallels the rise in related research publications. However, in the case of autoimmunity, more research is warranted to support the potential beneficial effects of Nrf2 modulation in each disease.

## 1. Introduction

Nuclear factor erythroid 2-related factor 2 (Nrf2, encoded by *Nfe2l2/NFE2L2*) is a transcription factor that coordinates the cellular responses to oxidative, electrophilic, and nitrosative stresses. Kelch-like ECH-associated protein 1 (Keap1) is a cysteine-rich protein that facilitates the rapid proteasomal degradation of Nrf2 under basal conditions. Upon exposure to reactive oxygen species, other stressors, or various natural and synthetic Nrf2-activating compounds, the sulfhydryl groups of Keap1 cysteines are modified, leading to allosteric modification of Keap1 that renders it unable to target Nrf2 for degradation. Thus, de novo transcribed and translated Nrf2 accumulates in the cell and enters the nucleus, where it recognizes specific sequences called antioxidant response elements (AREs) in the regulating regions of its target genes [1].

The first publication describing Nrf2 transcription factor appeared in the literature in 1994; since then, and especially after 2004, the number of publications on Nrf2 is ever increasing, having reached about 16,000 as of early 2020 [2]. The field of Nrf2 is expanding not only quantitatively but also qualitatively, with research varying from in vitro cell-based studies to model organisms with disrupted or enhanced Nrf2 signaling (e.g., *Caenorhabditis elegans* [3], *Drosophila* [4], zebrafish [5], mice [6,7], and rats [8]) to clinical trials with Nrf2-modulating compounds, such as sulforaphane [9,10] and C-28 methyl ester of 2-cyano-3,12-dioxoolean-1,9-dien-28-oic acid (CDDO-Me) [11,12]. Thus, knowledge of Nrf2 is increasing exponentially, and the pathway is showing promise in preclinical studies as a target for the prevention and treatment of various diseases, such as cancer [13], chronic kidney disease [14], chronic obstructive pulmonary disease (COPD) [15], neurodegenerative disorders [16], and diabetes [17]. Some of the identified Nrf2-activating compounds have already been used successfully in clinical trials to treat or prevent diseases [18]. The most prominent example is dimethyl fumarate (BG-12), which significantly reduces the rate of relapse, the rate of disability progression, and the number of magnetic resonance imaging (MRI)-detected lesions in patients with relapsing–remitting multiple sclerosis (MS) [19], and has received approval by the Food and Drug Administration (FDA) and the European Medicines Agency (EMA) for use in such patients. Sulforaphane in the form of broccoli sprout extracts has been shown to aid in the detoxification of airborne pollutants [20] as well as to reduce hepatic glucose production and to improve glycemic control in patients with uncontrolled type 2 diabetes [21]. Bardoxolone methyl has been shown to increase the glomerular filtration rate in patients with chronic kidney disease [22], even though one study was terminated early because of increased rate of cardiovascular events [22] for debatable reasons [23].

The role of the Nrf2 pathway has also been the focus of a series of studies on a variety of autoimmune diseases. The increased oxidative stress in tissues of subjects or model organisms with autoimmune diseases [24] along with the systemic inflammation inherent in autoimmunity has made the Nrf2 pathway an attractive potential target, not only because of its antioxidant cytoprotective role but also because activation of Nrf2 can block inflammatory responses at the transcriptional level by repressing upregulation of Il-6 and Il-1β in macrophages [25]. The higher prevalence of autoimmune diseases in women compared to men is well known. Consistent with that, female Nrf2 knockout mice develop systemic lupus erythematosus (SLE)-like autoimmune nephritis with aging [26] and show increased levels of circulating autoantibodies [27] as well as increased levels of markers of multiorgan autoimmune inflammation [28]. Absence of Nrf2 also exacerbates the phenotypes in mouse models of autoimmune inflammatory diseases, such as experimental autoimmune encephalomyelitis (EAE, a mouse model for MS) [29], rheumatoid arthritis (RA) [30], and systemic sclerosis (SS) [31]. Conversely, activation by genetic means (e.g., knockdown of its cytoplasmic inhibitor Keap1) has been shown to reduce systemic inflammation mainly by suppressing effector T-cell activities in scurfy mice that lack regulatory T-cells [32] and to reduce T-cell infiltration of the pancreatic islets and interferon (IFN)-γ levels, thereby delaying the onset of type 1 diabetes in nonobese diabetic (NOD) mice [33]. Furthermore, pharmacologic activation of Nrf2 signaling by TFM-735 [34], 3H-1,2-dithiole-3-thione [35], sulforaphane [36], dimethyl itaconate [37], or fumaric acid esters [38] ameliorates EAE. However, it should also be noted that the dosing of Nrf2 activators should be thoroughly assessed because a recent example shows that low doses can protect against inflammation, whereas high doses can cause detrimental effects [39].

The general increased interest in Nrf2 research is also reflected in the patents around the Nrf2 pathway; for example, a Google Patents search using the term “Nrf2 or Nfe2l2” results in about 2000 published patents in the last 10 years (search query: ((nrf2) OR (nfe2l2)) before:publication:20200528 after:publication:20100101 status:GRANT language:ENGLISH type:PATENT). The patent landscape around Nrf2 has been reviewed with reference to small-molecule Nrf2 activators for the periods 2012–2016 [40] and 2017–2020 [41]; these reviews show that considerable progress has been reported in the development and characterization of novel Nrf2 pathway activators. The main concerns are the specificity of each compound for the Nrf2 pathway and its applicability in various disease settings. Two older patent reviews had focused specifically on neurodegenerative diseases [42] and on sulforaphane [43].

In the present review of Nrf2-related patents, we took an unbiased approach to assess the broad patent landscape around Nrf2 using PatSeer Pro (Gridlogics, Pune, India), a web-based patent search, analytics, and landscaping platform. The goal was to obtain a general overview of Nrf2-related patents and to categorize their respective focus (e.g., activators, inhibitors, diagnostics, etc.) and broader topic (e.g., neurodegeneration, liver, kidney, lung or cardiovascular disease, cancer, etc.). The particular interest of our group in Nrf2 relates to thyroid physiology and disease [44]; in that regard, we have previously shown that (i) Nrf2 regulates the expression of thyroglobulin, the precursor of thyroid hormones [45,46]; (ii) constitutive genetic activation of Nrf2 causes thyroid enlargement (goiter) and mild hypothyroidism [47]; (iii) excess iodide exposure induces inflammatory, fibrosis, and autoimmune pathways along with Nrf2 signaling in the thyroid, which are accentuated by the absence of Nrf2 [48]; (iv) genetic variation in the *NFE2L2* promoter interacts with genetic variation in the promoter of a selenoprotein-encoding gene (*SELENOS*) to modulate the risk of autoimmune thyroiditis (Hashimoto’s disease) [49]; and (v) clinical use of the Nrf2 pathway activator sulforaphane is safe for thyroid hormonal and autoimmune status in humans [50]. Therefore, among the various topics emerging from the Nrf2-centered patent search, we focused primarily on patents relevant to autoimmune diseases, all the more so as they actually turned out to constitute a highly enriched topic among the patent search results.

## 2. Methods

We used the web-based tool PatSeer Pro (Gridlogics, Pune, India) to search for patents on Nrf2 from the period 2017–2020 based on the following search string: “Nrf2 OR Nfe2l2 OR Keap1” in the title and abstract of patents with publication dates from 1 January 2017 to 28 May 2020 with a legal status “active-pending” or “active-granted”. We limited the search to the last three years in order to focus on the most recently approved patents in the field as indicative of recent advances and current trends. This resulted in 594 patents, which after deduplication (i.e., retaining a single copy of each patent submitted to multiple agencies) yielded 509 unique patents. We then used the integrated tools of PatSeer Pro for text mining in the full text of patents, and the VizMap tool from the same platform to visualize the clusters of topics and themes of the Nrf2-related patents.

## 3. Results and Discussion

### 3.1. The 509 Unique Nrf2-Related Patents Published between January 2017 and May 2020

**Figure 1:**
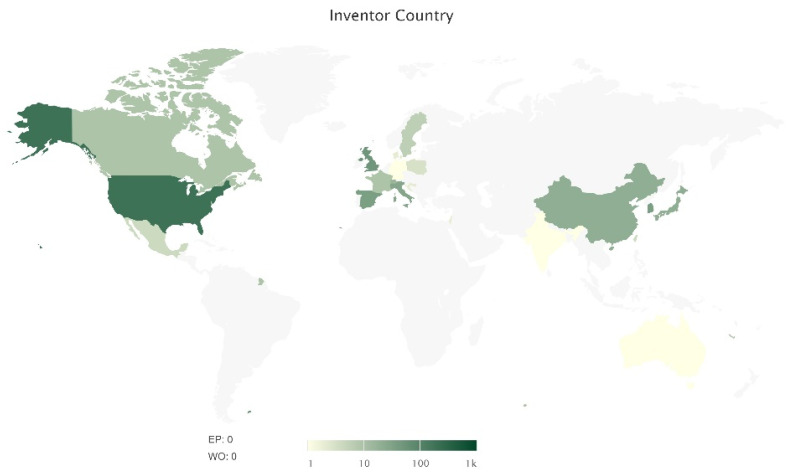
Inventor countries of Nrf2-related patents published from January 2017 to May 2020.

**Figure 2:**
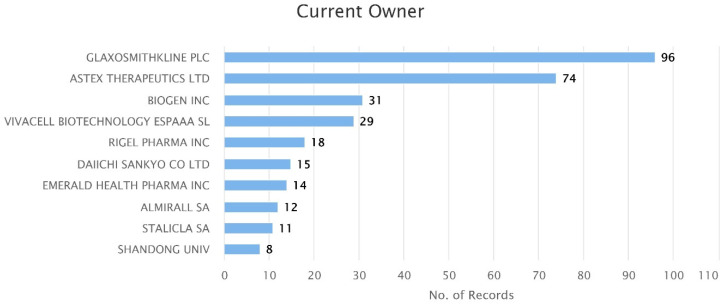
The top 10 owners of nuclear factor erythroid 2-related factor 2 (Nrf2) patents published from January 2017 to May 2020.

In the period between 1 January 2017 and 28 May 2020, 509 unique Nrf2-related patents were published. Geographically, the vast majority of the inventors of these patents were located in the USA, Europe, China, and Japan (Figure 1). This matches, to a large extent, our previous observations about the countries of origin of research publications on Nrf2 [2]. Table S1 lists all the patents along with basic information for each one (i.e., record number, title, current assignee, current owner, abstract, publication date, etc.). Notably, 9 out of 10 of the top current owners of Nrf2 patents are pharmaceutical companies (Figure 2), indicating that there is both research and commercial interest in Nrf2 pathway modulators.

### 3.2. Autoimmune and Oxidative Stress-Related Pathologies Are Main Topics of Nrf2-Related Patents

**Figure 3:**
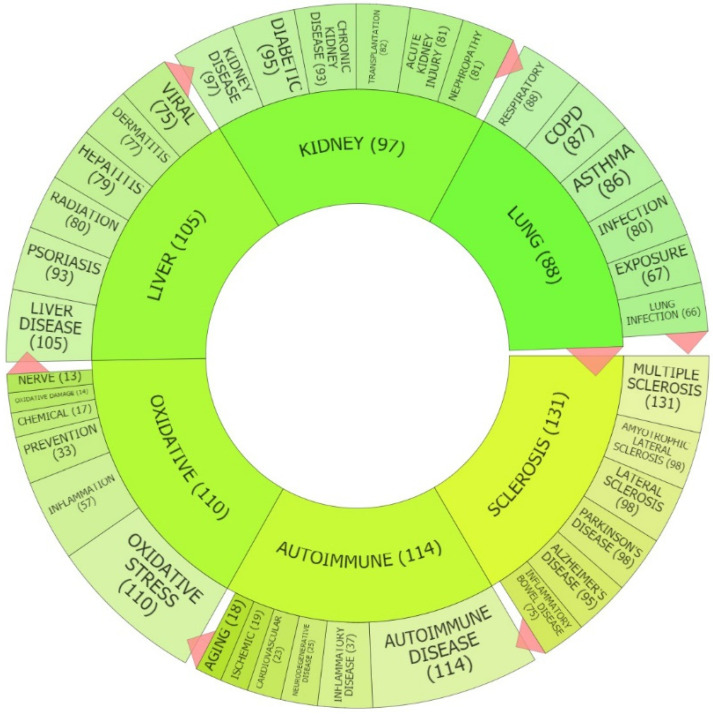
Clustering of the most enriched topics extracted from Kelch-like ECH-associated protein 1 (Keap1)/Nrf2 patents published from January 2017 to May 2020 using the PatSeer Pro platform. The number next to each topic indicates the number of patents discussing this topic. The major topics are depicted closer to the center of the circle and their relevant subtopics on the outer periphery of the circle.

The most enriched topics in the published patents on Keap1/Nrf2 are highlighted in Figure 3. It is evident that most patents include references to “sclerosis” (131 instances), “autoimmune” diseases (114 instances), “oxidative” stress (110 instances), “liver” (105 instances), “kidney” (97 instances), and “lung” (88 instances). Patents associated with “sclerosis” mainly refer to neurodegenerative diseases, such as MS, amyotrophic lateral sclerosis, Parkinson’s disease, and Alzheimer’s disease, but also to inflammatory bowel disease (IBD, encompassing Crohn’s disease and ulcerative colitis). The “autoimmune” topic is broader and encompasses patents relevant to a variety of autoimmune and inflammatory diseases, including neurodegenerative, cardiovascular, and aging-related diseases. The “oxidative” stress topic is also very broad and includes Keap1/Nrf2-related patents on anything relevant to reactive oxygen species and inflammation. The other three major topics refer to specific organs: liver, kidney, and lung. This highlights the interest in applications of Nrf2 pathway modulators in liver-related diseases, such as hepatitis, induced by chemicals or viruses; kidney-related pathologies (including diabetic nephropathy, chronic kidney disease, kidney transplantation, and acute kidney injury); and lung diseases (including respiratory tract infections, asthma, chronic obstructive pulmonary disease, and lung exposure to chemicals). This brief overview of Nrf2 patents thus highlights the main systems and major disease pathophysiologies that the inventors target. Supplementary Table S2 includes all topics depicted in Figure 3, along with less prevalent ones as well as their subcategories that do not fit in Figure 3 due to space restrictions.

**Figure 4:**
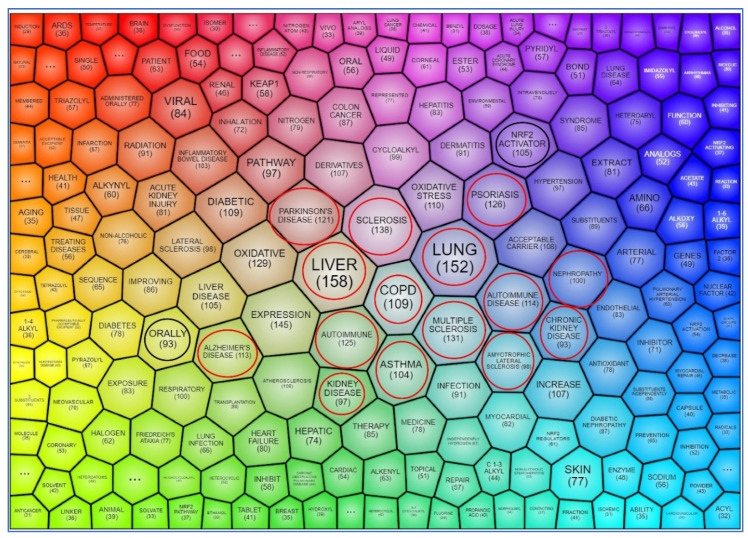
Clustering of the most common keywords found in Nrf2-related patents (in title, abstract, and full claims) published from January 2017 to May 2020, using PatSeer Pro. Numbers indicate the patents featuring each respective keyword. Red circles indicate keywords relevant to the topics shown in Figure 3, and black circles indicate Nrf2-modulating compounds and their route of administration.

These themes are also evident in the most common keywords found in patents (shown in Figure 4 with red circles and outlined in Supplementary Table S3). Even though the keyword analysis takes into account only the number of instances and does not categorize the terms, it highlights topics with reference to Nrf2-modulating-compounds (“Nrf2 activators”, 105 instances) and their route of administration (“orally”, 93 instances), both shown with black circles in Figure 4.

**Figure 5:**
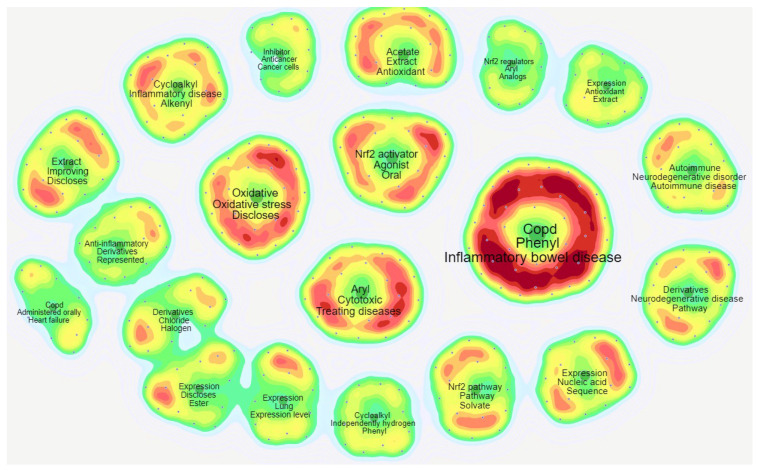
Top 20 themes emerging from Nrf2 patents published from January 2017 to May 2020 using the PatSeer Pro themes clustering tool (landscape mode). Each small dot represents a single patent. The gradient of colors from red to yellow to green indicates high, medium, or low co-occurrence, respectively, of the relevant themes in the specific group of patents.

An alternative clustering of the main themes emerging from Nrf2-related patents is “themes clustering”, whereby PatSeer Pro generates themes based on co-occurrence of keywords and topics. The top 20 enriched themes in Nrf2-related patents are depicted in Figure 5. Here, familiar terms encountered in topics clustering (Figure 3) are grouped in the same theme. For instance, “COPD”, “inflammatory bowel disease”, and “phenyl” are in the same theme, referring to the use of Nrf2 modulators to treat these diseases. “Nrf2 activator”, “agonist”, and “oral” form another theme that highlights the use of orally administered Nrf2 pathway activators as chemoprevention or treatment. Another interesting theme includes “inhibitor”, “anticancer”, and “cancer cells”, indicating the potential use of Nrf2 inhibitors in cancer treatment.

The aforementioned main topics and themes that emerged from the unbiased patent review, keyword analysis, and themes clustering, reflected very well the historical evolution of the Nrf2 research field as recently documented in an unbiased bibliometric analysis [2]. As a major regulator of the phase 2 detoxification response, Nrf2 has been studied early and extensively in the liver [8,51,52] and later in the lung [53,54,55] and in the kidney [56,57]. Strong interest in Nrf2 as a target for neurodegenerative diseases is linked to the high sensitivity of neurons to oxidative stress and the general unmet medical need in this disease area [58,59]. Lastly, interest in Nrf2 activators to bolster antioxidant capacity and detoxification for chemoprevention of cancer and other oxidative stress-related diseases historically preceded interest in Nrf2 inhibitors for the treatment of established cancers with an activated Nrf2 pathway [60,61,62,63].

### 3.3. Overview of Nrf2-Related Patents in Autoimmune Diseases

Autoimmune diseases emerged as one of the major topics (Figure 3) and themes (Figure 5) from the text mining and clustering analysis, yet they have not been previously examined in detail as a specific disease focus area of Nrf2-related patents. We therefore discuss these patents in more detail. Patent US10647697B2 focuses on heterocyclic compounds that can potentially activate the Nrf2 pathway for the treatment of psoriasis and MS. The inventors highlight two animal models: mice with severe combined immunodeficiency (SCID) transplanted with skin from a human psoriatic volunteer as a model relevant to psoriasis and mice with EAE as a model of autoimmune disease relevant to MS. Both these models can be treated with Nrf2 activators to assess their efficacy in the respective disorders. An example of the compounds tested in these patents is tert-butyl (E)-3-(5-oxo-4-(pyridin-3-yl)-4,5-dihydro-1H-tetrazol-1-yl)acrylate, for which there has been no clinical trial yet. The activation of Nrf2 of the tested compounds is evaluated by an Nrf2 translocation assay that shows that Nrf2 enters the nucleus. However, the specificity of the compounds for the Nrf2 pathway has not been evaluated, for example, by also using them in Nrf2 knockout mice in the respective disease models.

Patent WO2020081446A1 refers to autoimmune diseases in general by invoking the inflammatory aspect of these diseases, and it uses pulmonary fibrosis as an example where chronic inflammation that can be of autoimmune origin leads to fibrosis (idiopathic pulmonary fibrosis). The inventors and authors of the original paper describe the use of CBR-470-1, an inhibitor of phosphoglycerate kinase 1 (PGK1), and other relevant compounds as Nrf2-activating agents. CBR-470-1 has not yet been used in clinical trials, but its activity appears to be dependent on Nrf2 based on cell-based assays where Nrf2 was downregulated by short hairpin RNA [64].

Patent US2020079759A1 also suggests Nrf2 activation as a means of treating autoimmune diseases, such as psoriasis, IBD, and MS, among other diseases, but it focuses mainly on aryl analogs as Nrf2 activators. This patent focuses more on the synthesis of a variety of such analogs and does not include any experiments to evaluate the clinical effectiveness of these compounds against the claimed diseases, nor does it address their specificity with relevance to the Nrf2 pathway.

Similarly, patent US2020071317A1 proposes ether-linked triazole compounds as Nrf2 activators with potential efficacy against autoimmune diseases. These compounds have been well documented to directly inhibit the Keap1–Nrf2 protein–protein interaction and specifically the interaction between Keap1 and the DLG motif of Nrf2 but not the ETGE motif [65]. There are no published preclinical or clinical studies so far using these compounds in an experimental or clinical disease setting.

In a similar context, other inventors (patent US20200039933A1) propose indoline and benzimidazole compounds that activate Nrf2 and hypoxia-inducible factor 1 (HIF-1) and inhibit histone deacetylase (HDAC) as treatment for a wide range of autoimmune diseases, including endocrine autoimmune diseases (e.g., Hashimoto’s thyroiditis, Addison’s diseae, and type 1 diabetes), but without focusing on specific experiments or clinical trials to support this concept. Indoline-based compounds have also recently been described to directly affect the Keap1–Nrf2 protein–protein interaction and to exert antioxidant and cytoprotective roles in cell line-based experiments [66].

Another patent (WO2019104030A1) describes tetrahydronaphthalene derivatives as Nrf2 activators with potential utility against a variety of autoimmune diseases, such as IBD, RA, SLE, MS, type 1 diabetes, SS, and Sjögren’s syndrome (SjS). Again, this patent does not document specific supportive experiments for any of these suggested diseases, with the claims based rather on the general role of Nrf2 in reducing chronic inflammation and its potential benefit for autoimmune diseases in general. Naphthalene-based Nrf2 activators have been shown to effectively upregulate Nrf2 signaling in cell lines [67,68], supposedly by directly inhibiting the Keap1–Nrf2 protein–protein interaction. There is no evidence of clinical trials in progress using these compounds.

Other compounds that activate Nrf2 with potential therapeutic effect against autoimmune diseases are substituted hydroxystilbenes (patent US20190117589A1). Hydroxystilbenes are related to resveratrol and have been previously described to reduce the inflammatory response [69], although not necessarily through the Nrf2 pathway. Based on previous studies that have shown the role of Nrf2 in vitiligo [70,71] and in a variety of other autoimmune diseases (EAE, RA, MS, and psoriasis, reviewed in [72]), the inventors suggest a role of these compounds against autoimmune diseases through Nrf2 activation. It has been suggested that hydroxystilbenes-related compounds may perturb the Keap1–Nrf2 protein–protein interaction [73], but this has not yet been clearly documented by mechanistic studies. The most popular among these compounds is resveratrol, which has been used in a variety of clinical settings including, but not limited to, diabetes [74], cancer [75], Alzheimer’s disease [76], and cardiovascular disease [77,78]. There has been interest in the potential beneficial effect of resveratrol on autoimmune diseases [79], but most of the relevant studies are preclinical. Specifically, resveratrol has been shown to maintain the integrity of the blood–brain barrier in experimental autoimmune encephalomyelitis in mice [80], to confer neuroprotection in a mouse model of multiple sclerosis [81], to prevent type 1 diabetes in nonobese diabetic mice [82], and to reduce inflammation in a rheumatoid arthritis mouse model [83]. Clinical studies on the use of resveratrol against autoimmune diseases are also emerging in rheumatoid arthritis [84] and in type 1 diabetes [85].

Another patent (WO2018181345A1) suggests a heterocyclic compound and its derivatives of the category of noncondensed isoquinolines as an Nrf2 activator with potential action against a wide range of autoimmune diseases (MA, RA, SLE, SS, autoimmune hepatitis, type 1 diabetes, and IBD). Isoquinolines-based Nrf2 pathway activators also appear to interfere directly with the Keap1–Nrf2 protein–protein interaction [86] but it is not completely clear if they interfere with other pathways as well.

In the same context, other suggested Nrf2 pathway activators have been proposed as an effective regimen against most of the previously mentioned autoimmune diseases: bisaryl amides (US20190330238A1), 3-oxo-1,4-diazepinyle compounds (WO2018109648A1), bisaryl heterocycles (US20200062781A1), 3-(2,3-dihydro-1h-inden-5-yl)propanoic acid derivatives (WO2018104766A1), biaryl pyrazoles (US10364256B2), arylcyclohexyl pyrazoles (US10351530B2), 3-carboxylic acid pyrroles (US20200031820A1), as well as a variety of prodrugs (US20190367472A1). In these cases as well, the claims are rather generic; in vivo efficacy has not been demonstrated, and specificity for Nrf2 remains to be proven.

## 4. Conclusions

The main conclusions from the present patent review (2017–2020) of the Keap1/Nrf2 pathway using PatSeer Pro are the following: (i) PatSeer Pro is a user-friendly, rapid, and efficient web-based tool to conduct a patent search, analyze the search results, map the relevant patent landscape, and extract the respective topics and themes. As it is optimized for patent landscaping in any field, some of its limitations with relevance to life sciences include the inability to export a big variety of customized images and to identify and categorize the existing evidence for the claims of each patent (basic research studies, randomized clinical trials, etc.). (ii) Translational efforts around Nrf2 tend to stem from the same countries that have strong academic traditions in Nrf2 research, as evidenced by published literature. (iii) The predominant topics and themes in the Nrf2-related patent landscape reflect the most investigated clinical areas (i.e., autoimmunity, neurodegeneration, liver, lung, and kidney diseases) as well as the chronological emergence of concepts in Nrf2-focused academic research (i.e., oral Nrf2 activators initially for chemoprevention). (iv) The majority of the patents related to Nrf2 and autoimmune diseases from 2017–2020 focus on the use of nonelectrophilic Nrf2 pathway activators, which are shown or postulated to directly inhibit the Keap1–Nrf2 protein–protein interaction. This probably reflects the recent advances in the identification and evaluation of such Nrf2 pathway activators that do not react with the Keap1 sulfhydryl cysteine residues as is the case for electrophilic Nrf2 activators that are widely used in preclinical and clinical settings, such as sulforaphane, CDDO-Im (2-Cyano-3,12-dioxooleana-1,9-dien-28-imidazolide), bardoxolone, and dimethylfumarate. In this context, all patents that refer to Keap1 also refer to Nrf2 with reference to activation of this pathway by the aforementioned compounds. (v) Although autoimmune diseases represent the main area of translational interest for Nrf2 modulators, few of the relevant patents include sufficient experimental evidence to strongly support specific compounds as Nrf2 modulators with utility for respective autoimmune diseases. Thus, as basic and translational research on Nrf2 expands and extends to new disease areas, more emphasis should be placed on demonstrating the efficacy of Nrf2 modulators in each specific preclinical setting by utilizing appropriate respective models of disease. Lastly, although there is some evidence for most of the described Nrf2 pathway activators, at least from in vitro studies, in order to support the view that some of the protective effects are dependent on Nrf2, further research is warranted to describe the specificity of these compounds with respect to exclusively (or primarily) activating the Nrf2 pathway as opposed to modulating other molecular pathways in parallel. Such research is important because knowledge of the potential parallel or intersecting pathways that these compounds activate or inhibit can be valuable both before translating the basic research results in clinical trials to ensure patient safety as well as afterwards to help guide the explanation of the clinical outcomes.

## Supplementary Materials

The following are available online at https://www.mdpi.com/2076-3921/9/11/1138/s1. Table S1: Nrf2 patent list. Table S2: Keywords found in Nrf2 patents. Table S3: Topics generated from Nrf2 patents.

## Abbreviations

ARE	antioxidant response element
CDDO-Me	C-28 methyl ester of 2-cyano-3,12-dioxoolean-1,9-dien-28-oic acid
COPD	chronic obstructive pulmonary disease
EAE	experimental autoimmune encephalomyelitis
HIF1	hypoxia-inducible factor 1
IBD	inflammatory bowel disease
Keap1	Kelch-like ECH-associated protein 1
MS	multiple sclerosis
Nfe2l2	nuclear factor erythroid 2-like factor 2
Nrf2	nuclear factor erythroid 2-related factor 2
PGK1	phosphoglycerate kinase 1
RA	rheumatoid arthritis
SCID	severe combined immunodeficiency
SjS	Sjögren’s syndrome
SLE	systemic lupus erythematosus

## References

[1] Yamamoto M., Kensler T.W., Motohashi H. (2018). The KEAP1-NRF2 System: A Thiol-Based Sensor-Effector Apparatus for Maintaining Redox Homeostasis. Physiol. Rev..

[2] Paunkov A., Chartoumpekis D.V., Ziros P.G., Sykiotis G.P. (2019). A Bibliometric Review of the Keap1/Nrf2 Pathway and its Related Antioxidant Compounds. Antioxidants.

[3] Blackwell T.K., Steinbaugh M.J., Hourihan J.M., Ewald C.Y., Isik M. (2015). SKN-1/Nrf, stress responses, and aging in Caenorhabditis elegans. Free Radic. Biol. Med..

[4] Sykiotis G.P., Bohmann D. (2008). Keap1/Nrf2 signaling regulates oxidative stress tolerance and lifespan in Drosophila. Dev. Cell.

[5] Mukaigasa K., Tsujita T., Nguyen V.T., Li L., Yagi H., Fuse Y., Nakajima-Takagi Y., Kato K., Yamamoto M., Kobayashi M. (2018). Nrf2 activation attenuates genetic endoplasmic reticulum stress induced by a mutation in the phosphomannomutase 2 gene in zebrafish. Proc. Natl. Acad. Sci. USA.

[6] Itoh K., Chiba T., Takahashi S., Ishii T., Igarashi K., Katoh Y., Oyake T., Hayashi N., Satoh K., Hatayama I. (1997). An Nrf2/small Maf heterodimer mediates the induction of phase II detoxifying enzyme genes through antioxidant response elements. Biochem. Biophys. Res. Commun..

[7] Okawa H., Motohashi H., Kobayashi A., Aburatani H., Kensler T.W., Yamamoto M. (2006). Hepatocyte-specific deletion of the keap1 gene activates Nrf2 and confers potent resistance against acute drug toxicity. Biochem. Biophys. Res. Commun..

[8] Taguchi K., Takaku M., Egner P.A., Morita M., Kaneko T., Mashimo T., Kensler T.W., Yamamoto M. (2016). Generation of a New Model Rat: Nrf2 Knockout Rats Are Sensitive to Aflatoxin B1 Toxicity. Toxicol. Sci..

[9] Chen J.G., Johnson J., Egner P., Ng D., Zhu J., Wang J.B., Xue X.F., Sun Y., Zhang Y.H., Lu L.L. (2019). Dose-dependent detoxication of the airborne pollutant benzene in a randomized trial of broccoli sprout beverage in Qidong, China. Am. J. Clin. Nutr..

[10] Yagishita Y., Fahey J.W., Dinkova-Kostova A.T., Kensler T.W. (2019). Broccoli or Sulforaphane: Is It the Source or Dose That Matters?. Molecules.

[11] De Zeeuw D., Akizawa T., Audhya P., Bakris G.L., Chin M., Christ-Schmidt H., Goldsberry A., Houser M., Krauth M., Lambers Heerspink H.J. (2013). Bardoxolone methyl in type 2 diabetes and stage 4 chronic kidney disease. N. Engl. J. Med..

[12] Kanda H., Yamawaki K. (2020). Bardoxolone methyl: Drug development for diabetic kidney disease. Clin. Exp. Nephrol..

[13] Kensler T.W., Egner P.A., Agyeman A.S., Visvanathan K., Groopman J.D., Chen J.G., Chen T.Y., Fahey J.W., Talalay P. (2013). Keap1-nrf2 signaling: A target for cancer prevention by sulforaphane. Top. Curr. Chem..

[14] Nezu M., Suzuki N., Yamamoto M. (2017). Targeting the KEAP1-NRF2 System to Prevent Kidney Disease Progression. Am. J. Nephrol..

[15] Reddy N.M., Potteti H.R., Mariani T.J., Biswal S., Reddy S.P. (2011). Conditional deletion of Nrf2 in airway epithelium exacerbates acute lung injury and impairs the resolution of inflammation. Am. J. Respir. Cell Mol. Biol..

[16] Johnson D.A., Johnson J.A. (2015). Nrf2--a therapeutic target for the treatment of neurodegenerative diseases. Free Radic. Biol. Med..

[17] Chartoumpekis D.V., Kensler T.W. (2013). New player on an old field; the keap1/Nrf2 pathway as a target for treatment of type 2 diabetes and metabolic syndrome. Curr. Diabetes Rev..

[18] Cuadrado A., Rojo A.I., Wells G., Hayes J.D., Cousin S.P., Rumsey W.L., Attucks O.C., Franklin S., Levonen A.L., Kensler T.W. (2019). Therapeutic targeting of the NRF2 and KEAP1 partnership in chronic diseases. Nat. Rev. Drug Discov..

[19] Gold R., Kappos L., Arnold D.L., Bar-Or A., Giovannoni G., Selmaj K., Tornatore C., Sweetser M.T., Yang M., Sheikh S.I. (2012). Placebo-controlled phase 3 study of oral BG-12 for relapsing multiple sclerosis. N. Engl. J. Med..

[20] Egner P.A., Chen J.G., Zarth A.T., Ng D.K., Wang J.B., Kensler K.H., Jacobson L.P., Munoz A., Johnson J.L., Groopman J.D. (2014). Rapid and sustainable detoxication of airborne pollutants by broccoli sprout beverage: Results of a randomized clinical trial in China. Cancer Prev. Res..

[21] Axelsson A.S., Tubbs E., Mecham B., Chacko S., Nenonen H.A., Tang Y., Fahey J.W., Derry J.M.J., Wollheim C.B., Wierup N. (2017). Sulforaphane reduces hepatic glucose production and improves glucose control in patients with type 2 diabetes. Sci. Transl. Med..

[22] Nangaku M., Kanda H., Takama H., Ichikawa T., Hase H., Akizawa T. (2020). Randomized Clinical Trial on the Effect of Bardoxolone Methyl on GFR in Diabetic Kidney Disease Patients (TSUBAKI Study). Kidney Int. Rep..

[23] Chartoumpekis D.V., Sykiotis G.P. (2014). Bardoxolone methyl in type 2 diabetes and advanced chronic kidney disease. N. Engl. J. Med..

[24] Smallwood M.J., Nissim A., Knight A.R., Whiteman M., Haigh R., Winyard P.G. (2018). Oxidative stress in autoimmune rheumatic diseases. Free Radic. Biol. Med..

[25] Kobayashi E.H., Suzuki T., Funayama R., Nagashima T., Hayashi M., Sekine H., Tanaka N., Moriguchi T., Motohashi H., Nakayama K. (2016). Nrf2 suppresses macrophage inflammatory response by blocking proinflammatory cytokine transcription. Nat. Commun..

[26] Yoh K., Itoh K., Enomoto A., Hirayama A., Yamaguchi N., Kobayashi M., Morito N., Koyama A., Yamamoto M., Takahashi S. (2001). Nrf2-deficient female mice develop lupus-like autoimmune nephritis. Kidney Int..

[27] Li J., Stein T.D., Johnson J.A. (2004). Genetic dissection of systemic autoimmune disease in Nrf2-deficient mice. Physiol. Genom..

[28] Ma Q., Battelli L., Hubbs A.F. (2006). Multiorgan autoimmune inflammation, enhanced lymphoproliferation, and impaired homeostasis of reactive oxygen species in mice lacking the antioxidant-activated transcription factor Nrf2. Am. J. Pathol..

[29] Johnson D.A., Amirahmadi S., Ward C., Fabry Z., Johnson J.A. (2010). The absence of the pro-antioxidant transcription factor Nrf2 exacerbates experimental autoimmune encephalomyelitis. Toxicol. Sci..

[30] Maicas N., Ferrandiz M.L., Brines R., Ibanez L., Cuadrado A., Koenders M.I., van den Berg W.B., Alcaraz M.J. (2011). Deficiency of Nrf2 accelerates the effector phase of arthritis and aggravates joint disease. Antioxid. Redox Signal..

[31] Kavian N., Mehlal S., Jeljeli M., Saidu N.E.B., Nicco C., Cerles O., Chouzenoux S., Cauvet A., Camus C., Ait-Djoudi M. (2018). The Nrf2-Antioxidant Response Element Signaling Pathway Controls Fibrosis and Autoimmunity in Scleroderma. Front. Immunol..

[32] Suzuki T., Murakami S., Biswal S.S., Sakaguchi S., Harigae H., Yamamoto M., Motohashi H. (2017). Systemic Activation of NRF2 Alleviates Lethal Autoimmune Inflammation in Scurfy Mice. Mol. Cell. Biol..

[33] Yagishita Y., Uruno A., Chartoumpekis D.V., Kensler T.W., Yamamoto M. (2019). Nrf2 represses the onset of type 1 diabetes in non-obese diabetic mice. J. Endocrinol..

[34] Higashi C., Kawaji A., Tsuda N., Hayashi M., Saito R., Yagishita Y., Suzuki T., Uruno A., Nakamura M., Nakao K. (2017). The novel Nrf2 inducer TFM-735 ameliorates experimental autoimmune encephalomyelitis in mice. Eur. J. Pharmacol..

[35] Kuo P.C., Brown D.A., Scofield B.A., Yu I.C., Chang F.L., Wang P.Y., Yen J.H. (2016). 3H-1,2-dithiole-3-thione as a novel therapeutic agent for the treatment of experimental autoimmune encephalomyelitis. Brain Behav. Immun..

[36] Li B., Cui W., Liu J., Li R., Liu Q., Xie X.H., Ge X.L., Zhang J., Song X.J., Wang Y. (2013). Sulforaphane ameliorates the development of experimental autoimmune encephalomyelitis by antagonizing oxidative stress and Th17-related inflammation in mice. Exp. Neurol..

[37] Kuo P.C., Weng W.T., Scofield B.A., Paraiso H.C., Brown D.A., Wang P.Y., Yu I.C., Yen J.H. (2020). Dimethyl itaconate, an itaconate derivative, exhibits immunomodulatory effects on neuroinflammation in experimental autoimmune encephalomyelitis. J. Neuroinflamm..

[38] Linker R.A., Lee D.H., Ryan S., van Dam A.M., Conrad R., Bista P., Zeng W., Hronowsky X., Buko A., Chollate S. (2011). Fumaric acid esters exert neuroprotective effects in neuroinflammation via activation of the Nrf2 antioxidant pathway. Brain.

[39] Muri J., Wolleb H., Broz P., Carreira E.M., Kopf M. (2020). Electrophilic Nrf2 activators and itaconate inhibit inflammation at low dose and promote IL-1beta production and inflammatory apoptosis at high dose. Redox Biol..

[40] Sun H., Zhu J., Lin H., Gu K., Feng F. (2017). Recent progress in the development of small molecule Nrf2 modulators: A patent review (2012–2016). Expert Opin. Ther. Pat..

[41] Zhou H., Wang Y., You Q., Jiang Z. (2020). Recent progress in the development of small molecule Nrf2 activators: A patent review (2017-present). Expert Opin. Ther. Pat..

[42] Joshi G., Johnson J.A. (2012). The Nrf2-ARE pathway: A valuable therapeutic target for the treatment of neurodegenerative diseases. Recent Pat. CNS Drug Discov..

[43] De Figueiredo S.M., Binda N.S., Nogueira-Machado J.A., Vieira-Filho S.A., Caligiorne R.B. (2015). The antioxidant properties of organosulfur compounds (sulforaphane). Recent Pat. Endocr. Metab. Immune Drug Discov..

[44] Christina Thanas P.G.Z., Chartoumpekis D.V., Renaud C.O., Sykiotis G.P. (2020). The Keap1/Nrf2 signaling pathway in the thyroid—2020 update. Antioxidants.

[45] Ziros P.G., Habeos I.G., Chartoumpekis D.V., Ntalampyra E., Somm E., Renaud C.O., Bongiovanni M., Trougakos I.P., Yamamoto M., Kensler T.W. (2018). NFE2-Related Transcription Factor 2 Coordinates Antioxidant Defense with Thyroglobulin Production and Iodination in the Thyroid Gland. Thyroid.

[46] Matana A., Ziros P.G., Chartoumpekis D.V., Renaud C.O., Polasek O., Hayward C., Zemunik T., Sykiotis G.P. (2020). Rare and common genetic variations in the Keap1/Nrf2 antioxidant response pathway impact thyroglobulin gene expression and circulating levels, respectively. Biochem. Pharmacol..

[47] Ziros P.G., Renaud C.O., Chartoumpekis D.V., Bongiovanni M., Habeos I.G., Liao X.H., Refetoff S., Kopp P.A., Brix K., Sykiotis G.P. (2020). Mice Hypomorphic for Keap1, a Negative Regulator of the Nrf2 Antioxidant Response, Show Age-Dependent Diffuse Goiter with Elevated Thyrotropin Levels. Thyroid.

[48] Chartoumpekis D.V., Ziros P.G., Georgakopoulos-Soares I., Smith A.A.T., Marques A.C., Ibberson M., Kopp P.A., Habeos I., Trougakos I.P., Khoo N.K.H. (2020). The Transcriptomic Response of the Murine Thyroid Gland to Iodide Overload and the Role of the Nrf2 Antioxidant System. Antioxidants.

[49] Santos L.R., Duraes C., Ziros P.G., Pestana A., Esteves C., Neves C., Carvalho D., Bongiovanni M., Renaud C.O., Chartoumpekis D.V. (2019). Interaction of Genetic Variations in NFE2L2 and SELENOS Modulates the Risk of Hashimoto’s Thyroiditis. Thyroid.

[50] Chartoumpekis D.V., Ziros P.G., Chen J.G., Groopman J.D., Kensler T.W., Sykiotis G.P. (2019). Broccoli sprout beverage is safe for thyroid hormonal and autoimmune status: Results of a 12-week randomized trial. Food Chem. Toxicol..

[51] Osburn W.O., Yates M.S., Dolan P.D., Chen S., Liby K.T., Sporn M.B., Taguchi K., Yamamoto M., Kensler T.W. (2008). Genetic or pharmacologic amplification of nrf2 signaling inhibits acute inflammatory liver injury in mice. Toxicol. Sci..

[52] Enomoto A., Itoh K., Nagayoshi E., Haruta J., Kimura T., O’Connor T., Harada T., Yamamoto M. (2001). High sensitivity of Nrf2 knockout mice to acetaminophen hepatotoxicity associated with decreased expression of ARE-regulated drug metabolizing enzymes and antioxidant genes. Toxicol. Sci..

[53] Kikuchi N., Ishii Y., Morishima Y., Yageta Y., Haraguchi N., Itoh K., Yamamoto M., Hizawa N. (2010). Nrf2 protects against pulmonary fibrosis by regulating the lung oxidant level and Th1/Th2 balance. Respir. Res..

[54] Sussan T.E., Rangasamy T., Blake D.J., Malhotra D., El-Haddad H., Bedja D., Yates M.S., Kombairaju P., Yamamoto M., Liby K.T. (2009). Targeting Nrf2 with the triterpenoid CDDO-imidazolide attenuates cigarette smoke-induced emphysema and cardiac dysfunction in mice. Proc. Natl. Acad. Sci. USA.

[55] Reddy N.M., Kleeberger S.R., Cho H.Y., Yamamoto M., Kensler T.W., Biswal S., Reddy S.P. (2007). Deficiency in Nrf2-GSH signaling impairs type II cell growth and enhances sensitivity to oxidants. Am. J. Respir. Cell Mol. Biol..

[56] Liu M., Grigoryev D.N., Crow M.T., Haas M., Yamamoto M., Reddy S.P., Rabb H. (2009). Transcription factor Nrf2 is protective during ischemic and nephrotoxic acute kidney injury in mice. Kidney Int..

[57] Miyazaki Y., Shimizu A., Pastan I., Taguchi K., Naganuma E., Suzuki T., Hosoya T., Yokoo T., Saito A., Miyata T. (2014). Keap1 inhibition attenuates glomerulosclerosis. Nephrol. Dial. Transplant..

[58] Joshi G., Gan K.A., Johnson D.A., Johnson J.A. (2015). Increased Alzheimer’s disease-like pathology in the APP/ PS1DeltaE9 mouse model lacking Nrf2 through modulation of autophagy. Neurobiol. Aging.

[59] Simpson D.S.A., Oliver P.L. (2020). ROS Generation in Microglia: Understanding Oxidative Stress and Inflammation in Neurodegenerative Disease. Antioxidants.

[60] Slocum S.L., Kensler T.W. (2011). Nrf2: Control of sensitivity to carcinogens. Arch. Toxicol..

[61] Yates M.S., Kwak M.K., Egner P.A., Groopman J.D., Bodreddigari S., Sutter T.R., Baumgartner K.J., Roebuck B.D., Liby K.T., Yore M.M. (2006). Potent protection against aflatoxin-induced tumorigenesis through induction of Nrf2-regulated pathways by the triterpenoid 1-[2-cyano-3-,12-dioxooleana-1,9(11)-dien-28-oyl]imidazole. Cancer Res..

[62] Kansanen E., Kuosmanen S.M., Leinonen H., Levonen A.L. (2013). The Keap1-Nrf2 pathway: Mechanisms of activation and dysregulation in cancer. Redox Biol..

[63] Zhu J., Wang H., Chen F., Fu J., Xu Y., Hou Y., Kou H.H., Zhai C., Nelson M.B., Zhang Q. (2016). An overview of chemical inhibitors of the Nrf2-ARE signaling pathway and their potential applications in cancer therapy. Free Radic. Biol. Med..

[64] Bollong M.J., Lee G., Coukos J.S., Yun H., Zambaldo C., Chang J.W., Chin E.N., Ahmad I., Chatterjee A.K., Lairson L.L. (2018). A metabolite-derived protein modification integrates glycolysis with KEAP1-NRF2 signalling. Nature.

[65] Bertrand H.C., Schaap M., Baird L., Georgakopoulos N.D., Fowkes A., Thiollier C., Kachi H., Dinkova-Kostova A.T., Wells G. (2015). Design, Synthesis, and Evaluation of Triazole Derivatives That Induce Nrf2 Dependent Gene Products and Inhibit the Keap1-Nrf2 Protein-Protein Interaction. J. Med. Chem..

[66] Zhou H.S., Hu L.B., Zhang H., Shan W.X., Wang Y., Li X., Liu T., Zhao J., You Q.D., Jiang Z.Y. (2020). Design, Synthesis, and Structure-Activity Relationships of Indoline-Based Kelch-like ECH-Associated Protein 1-Nuclear Factor (Erythroid-Derived 2)-Like 2 (Keap1-Nrf2) Protein-Protein Interaction Inhibitors. J. Med. Chem..

[67] Jain A.D., Potteti H., Richardson B.G., Kingsley L., Luciano J.P., Ryuzoji A.F., Lee H., Krunic A., Mesecar A.D., Reddy S.P. (2015). Probing the structural requirements of non-electrophilic naphthalene-based Nrf2 activators. Eur. J. Med. Chem..

[68] Son T.G., Kawamoto E.M., Yu Q.S., Greig N.H., Mattson M.P., Camandola S. (2013). Naphthazarin protects against glutamate-induced neuronal death via activation of the Nrf2/ARE pathway. Biochem. Biophys. Res. Commun..

[69] Dang O., Navarro L., David M. (2004). Inhibition of lipopolysaccharide-induced interferon regulatory factor 3 activation and protection from septic shock by hydroxystilbenes. Shock.

[70] Jian Z., Li K., Song P., Zhu G., Zhu L., Cui T., Liu B., Tang L., Wang X., Wang G. (2014). Impaired activation of the Nrf2-ARE signaling pathway undermines H_2_O_2_-induced oxidative stress response: A possible mechanism for melanocyte degeneration in vitiligo. J. Investig. Dermatol..

[71] Ma J., Li S., Zhu L., Guo S., Yi X., Cui T., He Y., Chang Y., Liu B., Li C. (2018). Baicalein protects human vitiligo melanocytes from oxidative stress through activation of NF-E2-related factor2 (Nrf2) signaling pathway. Free Radic. Biol. Med..

[72] Cuadrado A., Manda G., Hassan A., Alcaraz M.J., Barbas C., Daiber A., Ghezzi P., Leon R., Lopez M.G., Oliva B. (2018). Transcription Factor NRF2 as a Therapeutic Target for Chronic Diseases: A Systems Medicine Approach. Pharmacol. Rev..

[73] Bhakkiyalakshmi E., Dineshkumar K., Karthik S., Sireesh D., Hopper W., Paulmurugan R., Ramkumar K.M. (2016). Pterostilbene-mediated Nrf2 activation: Mechanistic insights on Keap1:Nrf2 interface. Bioorg. Med. Chem..

[74] Bhatt J.K., Thomas S., Nanjan M.J. (2012). Resveratrol supplementation improves glycemic control in type 2 diabetes mellitus. Nutr. Res..

[75] Howells L.M., Berry D.P., Elliott P.J., Jacobson E.W., Hoffmann E., Hegarty B., Brown K., Steward W.P., Gescher A.J. (2011). Phase I randomized, double-blind pilot study of micronized resveratrol (SRT501) in patients with hepatic metastases--safety, pharmacokinetics, and pharmacodynamics. Cancer Prev. Res..

[76] Turner R.S., Thomas R.G., Craft S., van Dyck C.H., Mintzer J., Reynolds B.A., Brewer J.B., Rissman R.A., Raman R., Aisen P.S. (2015). A randomized, double-blind, placebo-controlled trial of resveratrol for Alzheimer disease. Neurology.

[77] Berman A.Y., Motechin R.A., Wiesenfeld M.Y., Holz M.K. (2017). The therapeutic potential of resveratrol: A review of clinical trials. NPJ Precis. Oncol..

[78] Tome-Carneiro J., Gonzalvez M., Larrosa M., Yanez-Gascon M.J., Garcia-Almagro F.J., Ruiz-Ros J.A., Garcia-Conesa M.T., Tomas-Barberan F.A., Espin J.C. (2012). One-year consumption of a grape nutraceutical containing resveratrol improves the inflammatory and fibrinolytic status of patients in primary prevention of cardiovascular disease. Am. J. Cardiol..

[79] Oliveira A.L.B., Monteiro V.V.S., Navegantes-Lima K.C., Reis J.F., Gomes R.S., Rodrigues D.V.S., Gaspar S.L.F., Monteiro M.C. (2017). Resveratrol Role in Autoimmune Disease-A Mini-Review. Nutrients.

[80] Wang D., Li S.P., Fu J.S., Zhang S., Bai L., Guo L. (2016). Resveratrol defends blood-brain barrier integrity in experimental autoimmune encephalomyelitis mice. J. Neurophysiol..

[81] Fonseca-Kelly Z., Nassrallah M., Uribe J., Khan R.S., Dine K., Dutt M., Shindler K.S. (2012). Resveratrol neuroprotection in a chronic mouse model of multiple sclerosis. Front. Neurol..

[82] Lee S.M., Yang H., Tartar D.M., Gao B., Luo X., Ye S.Q., Zaghouani H., Fang D. (2011). Prevention and treatment of diabetes with resveratrol in a non-obese mouse model of type 1 diabetes. Diabetologia.

[83] Riveiro-Naveira R.R., Valcarcel-Ares M.N., Almonte-Becerril M., Vaamonde-Garcia C., Loureiro J., Hermida-Carballo L., Lopez-Pelaez E., Blanco F.J., Lopez-Armada M.J. (2016). Resveratrol lowers synovial hyperplasia, inflammatory markers and oxidative damage in an acute antigen-induced arthritis model. Rheumatology.

[84] Khojah H.M., Ahmed S., Abdel-Rahman M.S., Elhakeim E.H. (2018). Resveratrol as an effective adjuvant therapy in the management of rheumatoid arthritis: A clinical study. Clin. Rheumatol..

[85] Movahed A., Raj P., Nabipour I., Mahmoodi M., Ostovar A., Kalantarhormozi M., Netticadan T. (2020). Efficacy and Safety of Resveratrol in Type 1 Diabetes Patients: A Two-Month Preliminary Exploratory Trial. Nutrients.

[86] Lazzara P.R., David B.P., Ankireddy A., Richardson B.G., Dye K., Ratia K.M., Reddy S.P., Moore T.W. (2020). Isoquinoline Kelch-like ECH-Associated Protein 1-Nuclear Factor (Erythroid-Derived 2)-like 2 (KEAP1-NRF2) Inhibitors with High Metabolic Stability. J. Med. Chem..

